# Prevalence, Risk Factors, and Clinical Relevance of Fluoroquinolone-Resistant Organisms in Rectal Cultures: Should We Target Antibiotic Prophylaxis Prior to Prostate Biopsy?

**DOI:** 10.1155/2016/5392107

**Published:** 2016-03-01

**Authors:** J. Van Besien, P. Uvin, A. M. Van den Abeele, L. Merckx

**Affiliations:** ^1^Department of Urology, AZ Sint-Lucas, 9000 Ghent, Belgium; ^2^Department of Microbiology, AZ Sint-Lucas, 9000 Ghent, Belgium

## Abstract

The rise of infectious complications after prostate biopsy has been linked to the growing resistance of enterobacteria to fluoroquinolone (FQ) antibiotics. In this review, we investigated the potential benefit of targeted antibiotic prophylaxis based on rectal cultures prior to prostate biopsy. An electronic search for all related literature published in English was performed from April until June 2015 using the MEDLINE and EMBASE databases. Data were obtained regarding the true prevalence of FQ-resistant bacteria in the rectum of patients, the identification of those patients at risk of harbouring FQ-resistant bacteria, the risk of infectious complications after transrectal prostate biopsy in patients with FQ-resistant bacteria, and the effect of targeted prophylaxis. Although there is limited evidence that a targeted approach might be beneficial, we conclude that current studies on the use of rectal cultures in the prebiopsy setting have too many limitations and confounding variables to definitely accept this approach in clinical practice. Whether this methodology is useful in a certain region will greatly depend on local fluoroquinolone-resistance rates.

## 1. Introduction

Prostate cancer is the most common type of cancer in elderly males in Europe and is, only second after lung cancer, the leading cause of cancer death in men. Prostate biopsy is a well-established and very common procedure that is used worldwide for the diagnosis and risk stratification of prostate cancer. Ultrasound-guided transrectal prostate biopsies (TRPB) are most frequently used although some urologists prefer a perineal approach. No significant differences in cancer detection rate or infectious complications were found between the two approaches [1]. Pain and bleeding are common complications after TRPB. The risk of major complications (such as sepsis) however is very small. The incidence of infectious complications after TRPB varies among studies, with a reported hospitalization rate of 0–6.3% [2]. Also, it has been demonstrated that antimicrobial prophylaxis significantly reduces the risk of these infectious complications [3]. Despite the widespread use of prophylactic fluoroquinolone (FQ) antibiotics, several studies report an increase of infectious complications in recent years [4, 5]. Indeed, FQ-resistant (FQ-R) bacteria most commonly cause infectious complications after TRPB and the increased prevalence of these complications correlates with the steady increase of FQ-resistance [6].

The use of rectal cultures (by rectal swabs or stool samples) before TRPB has been suggested for different purposes: (1) to determine the true prevalence of FQ-R bacteria in the rectum of patients, (2) to identify patients who are at risk of harbouring FQ-R bacteria, (3) to establish whether there is higher prevalence of infectious complications after TRPB in patients with FQ-R bacteria, and (4) to serve as a guide for targeted prophylaxis [7–25]. We reviewed the literature investigating the use of rectal cultures in the prebiopsy setting in order to summarize the current evidence and to elucidate future research perspectives.

## 2. Methods

**Figure 1:**
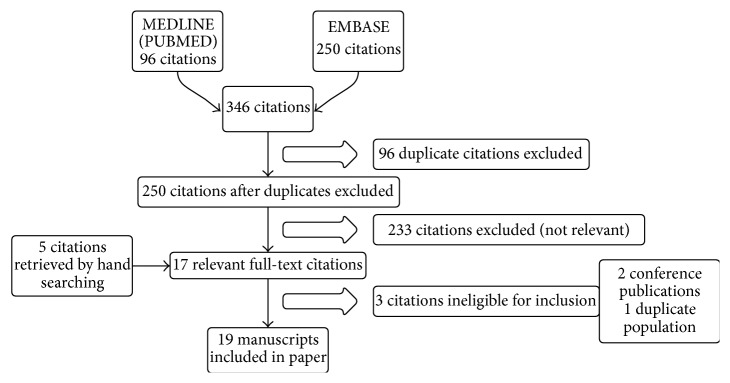
Flowchart of study selection.

An electronic search for all related literature published in English was performed from April until June 2015. The following databases were used: MEDLINE via PUBMED, EMBASE. The keywords rectal, culture, prostate, and biopsy were used in different combinations and with different synonyms. 346 papers were found and one author (JVB) screened all articles by title and abstract. All studies that reported sampling of prebiopsy rectal cultures and assessment of FQ sensitivity profiles were considered relevant. After exclusion of duplicate and irrelevant papers, 17 papers were retrieved in full text for formal review. A manual search of the references list of these articles revealed another five relevant articles. Three papers were ineligible for inclusion because of a duplicate population or because the abstract concerned a conference publication. Finally, 19 papers were withheld for this review. A flowchart of the study selection procedure is displayed in Figure 1.

Data were extracted from full-text articles and were entered into a database. The extracted data included the study type; the use of empirical or targeted therapy; the exact timing of the retrieval of the rectal cultures; the duration of prophylaxis; the prevalence, type, and grade of complications; the culture methodology; the descriptive characteristics and demographics of the included patients; the identification of bacterial species causing complications; the number of patients with FQ-R bacteria.

## 3. Results

### 3.1. Prevalence of Intrarectal FQ-R Bacteria in Patients Undergoing Prostate Biopsy

**Figure 2:**
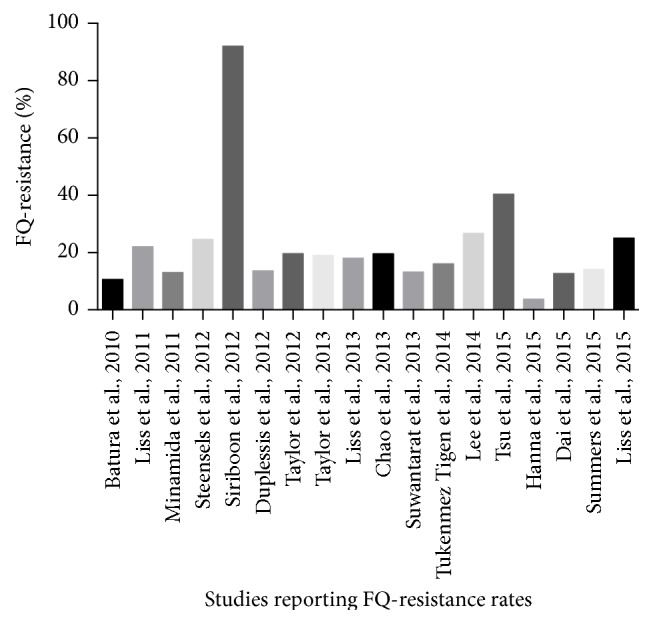
Prevalence of fluoroquinolone-resistance in rectal cultures prior to prostate biopsy.

The prevalence of FQ-R bacteria in the intestinal flora of patients undergoing TRPB has been well documented. The first study to describe this prevalence was performed by Batura et al. in the United Kingdom (UK) in 2010 [7]. Rectal swabs were collected from 445 patients and isolation of Gram-negative aerobic bacteria was performed using a selective agar (cystine lactose electrolyte deficient agar). Antimicrobial sensitivity of rectal bacteria to ciprofloxacin was determined by disk diffusion technique. In only 10.6% of these patients FQ-resistance was found. The risk of infectious complications was much higher in the group harbouring FQ-R organisms compared to the group with FQ-sensitive (FQ-S) organisms. Hereafter, various study groups repeated this study under varying circumstances and have described various prevalence rates [8–23, 25]. Prevalence rates are displayed in Figure 2. One study conducted in Bangkok described an extremely high prevalence rate of 92% for FQ-R organisms [11]. Other authors confirmed the higher FQ-R prevalence rate in Asian countries, compared to European and American studies. Lee et al. reported a FQ-R prevalence rate of 27% in Korea, while Tsu et al. described a FQ-R prevalence rate of 40% FQ in Hong Kong [19, 20]. Intriguingly, Minamida et al. found a FQ-R prevalence rate of only 13% in Japan [9]. In general, in studies from Asia, 43% of the patients were harbouring FQ-R bacteria in their cultures. In Europe, studies were performed in the UK in 2010, in Belgium in 2012, in Turkey in 2014, and again in the UK in 2015. FQ-R prevalence rates of, respectively, 10%, 25%, 16%, and 4% were reported. Summarized, FQ-R bacteria were found in 12% of the rectal cultures of European patients [7, 10, 18, 21]. In the United States of America studies were performed in California in 2011, 2012, 2013, and 2015; in Illinois in 2012 and 2013; in Ohio in 2013; in Washington in 2015; and in Utah in 2015 [8, 12, 13, 15–17, 22, 23, 25]. FQ-R rates differed from 12.7% in Washington to 25% in California [22, 25]. In Canada, a study conducted in Vancouver described a FQ-R prevalence rate of 19% [14]. In North America, a mean FQ-R prevalence rate of 19% was found.

### 3.2. Identification of Parameters to Define Patients at Risk of Rectal FQ-R Bacteria

Different patient characteristics have been examined to identify risk factors for harbouring FQ-R bacteria. These include age, body mass index (BMI), prostate-specific antigen (PSA), prior biopsies, prostate volume, American Urological Association symptom score, Charlson Comorbidity Index, Total Illness Burden Index for Prostate Cancer score, race, the presence of diabetes mellitus, a history of urinary tract infections, family members in health care, hospitalization for illness in the last 12 months, presence of prostate cancer, a history of FQ use, and a history of non-FQ antibiotics use. Ten studies used one or more of these characteristics to identify risk groups. Three studies were unable to detect statistically significant differences for any examined parameter [12, 15, 22]. A history of previous prostate biopsies created a difference in two studies without being statistically significant [8, 9] and a significant difference (*P* = 0.032) in one study [23]. Previous antibiotic exposure, in particular FQ exposure, was responsible for a significant difference in five studies (*P* < 0.01 [9]; *P* < 0.01 [10]; *P* value not specified [11]; *P* < 0.005 [14]; *P* = 0.04 [20]). The presence of diabetes mellitus produced an almost statistically significant difference in one study [8] and a statistically significant difference in another study (*P* = 0.02) [20]. A history of chronic prostatitis [10], recent urinary tract infection (UTI) [14], recent positive urinary culture [23], old(er) age [8], a higher PSA density [9], and presence of heart valve replacement [14] were responsible for statistically significant differences. BMI, American Urological Association symptom score, Charlson Comorbidity Index, Total Illness Burden Index for Prostate Cancer score, race, family members in health care, hospitalization for illness in the last 12 months, and presence of prostate cancer were no risk factors for harbouring FQ-R bacteria.

### 3.3. Presence of FQ-R Bacteria as a Risk Factor for the Development of Infectious Complications after Biopsy

In 13 studies, rectal cultures were taken immediately before prostate biopsy without influencing the choice of the prophylactic therapy [7–11, 14–21]. Two of these studies did not specify the complication rate in FQ-R versus FQ-S groups [16, 18]. Compiled results of the 11 other studies describe a complication rate of 7.9% in the FQ-R group (53 out of 666 patients). A very wide range in the complication rate was noted with a minimum of 0% in 2 studies [15, 21] and a maximum of 43% in another study [17]. The complication rate in the FQ-S group was only 1.6% (36 out of 2247 patients). Again, a wide range in the complication rate was found with a minimum of 0% in 4 studies [9–11, 15] and a maximum of 3.8% in another study [8]. Thus, after the use of FQ prophylaxis before prostate biopsy, there was a fivefold higher risk of infectious complications in the presence of FQ-resistance. The overall complication rate in these patients was 3% (89 out of 2913 patients) which is comparable to the rates reported in literature [2]. Results are summarized in Table 1.

### 3.4. Examining the Results of Targeted Prophylaxis

**Figure 3:**
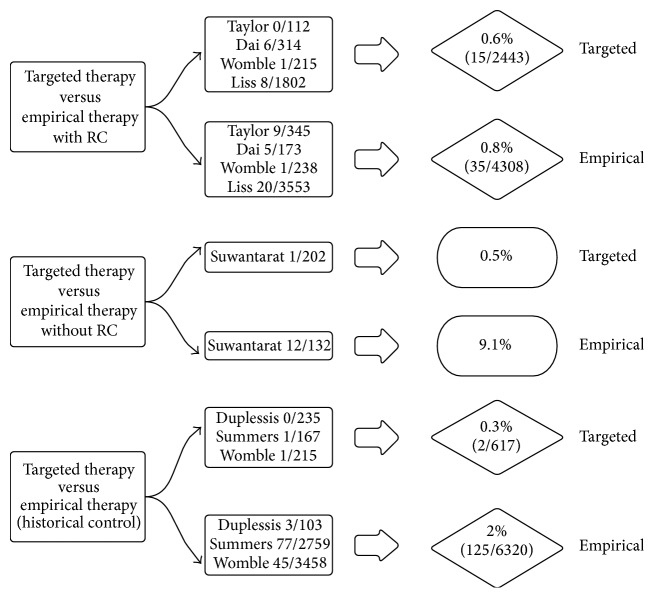
The risk of infectious complications after prostate biopsy in studies using targeted versus empirical antibiotic therapy.

Seven studies were found in which at least a part of the study population was offered targeted prophylaxis instead of empirical prophylaxis before TRPB. We discriminated between targeted unmodified and targeted modified prophylaxis. Targeted modified and unmodified prophylaxis are both based on the results of rectal cultures. The targeted unmodified patients are those patients with FQ sensitivity. The antibiotic prophylaxis was “unmodified” in this group. This means that it was the same prophylaxis as if no rectal cultures were taken. This is no empirical prophylaxis stricto sensu since FQ sensitivity is proven. The targeted modified patients are those patients with FQ-resistance. The antibiotic prophylaxis was “modified” meaning that it was not the same prophylaxis as if no rectal cultures were taken. A total of 3047 patients received targeted prophylaxis: 2248 patients targeted unmodified and 576 targeted modified prophylaxis. Complications were reported in a considerable part of the patients from the targeted group. The complication rate in the targeted unmodified group was 0.5%, while this was 0.8% in the targeted modified group [12, 13, 17, 22–25]. Four studies prospectively compared the results of the targeted approach to an empirical approach in which no rectal cultures were taken. In the empirical group, the complication rate was 0.8% (35 out of 4309 patients) while this was 0.6% in the targeted group (15 out of 2443 patients) [13, 22, 24, 25]. In one prospective trial, rectal cultures were taken immediately before biopsy in one group (empirical therapy group) and 1 month before biopsy in another group (targeted therapy group). The complication rate in the empirical therapy group was 9.1% while this was 0.5% in the targeted therapy group [17]. Three studies compared infectious complications in a prospective targeted therapy group to infectious complications in a retrospective (historical) control group. Two infectious complications were seen in 617 biopsies in the targeted therapy group (0.3%) compared to 125 infectious complications in 6320 patients in the empirical therapy group (2%) [12, 23, 24]. Results are summarized in Figure 3.

## 4. Discussion

The rise of infectious complications after prostate biopsy has been linked to the growing resistance of bacteria for FQ antibiotics. In 2010, statistical models predicted that by 2013 the rate of FQ-R* Escherichia coli* would be as high as 45% in populations with high FQ usage [26]. Despite this evidence, FQs are in most countries still the first-choice prophylactic antibiotics for prostate biopsies. To manage the problem of increased FQ-resistance, both broadening of the antibiotic prophylactic spectrum with the administration of a second antibiotic agent and targeted antibiotic prophylaxis have been attempted. Adding an antibiotic agent has been shown to reduce complications and hospital admissions in several studies [27, 28]. However, this approach predisposes to significant overtreatment in the majority of patients (i.e., those carrying FQ-S bacteria). The antibiotics that are used to augment therapy (mostly gentamicin or amikacin) are often used in salvage regimen in the treatment of extended spectrum beta lactamase (ESBL) bacteria that have increasing prevalence.

In this review, we tried to summarize the current evidence about the use of rectal cultures in the prebiopsy setting. FQ-R bacteria cause the majority of the complications after prostate biopsy. The ultimate goal of inclusion of rectal cultures in prostate biopsy protocols would be to target the antibiotic therapy for the individual patient in order to prevent more infectious complications. Another goal is to reduce the use of FQs when FQ-R bacteria are present and to avoid unnecessary administration of a second antibiotic agent. We are currently conducting a clinical trial to evaluate the potential advantages of targeted antibiotic prophylaxis based on rectal cultures, compared to empirical antibiotic prophylaxis. All procedures have been approved by the Ethical Review Board at AZ Sint-Lucas Hospital, Ghent, Belgium.

Targeting antibiotic prophylaxis starts with determining the prevalence of FQ-R bacteria in the rectum of patients undergoing transrectal biopsy. As described above, this prevalence depends greatly on the geographic location and is influenced by local antibiotic treatment policies. It is well known that unjudicious use of antibiotics in human and veterinary medicine and in agriculture may lead to extensive resistance rates. However, we must consider that there are other factors influencing these obvious differences in prevalence. Firstly, various culture methodologies are reported in different studies. Patients might have multiple* E. coli* strains at a time, both FQ-R and FQ-S ones. Different microbiological methodologies may lead to variable detection rates of resistant strains. Secondly, the exact timing of the sampling of the rectal culture might influence the reported prevalence rate. Studies examining the pharmacokinetics of oral FQ have shown excellent bioavailability (70–99%) with peak serum concentrations observed after 1 hour. Moreover, it was demonstrated that these concentrations were even higher in tissue than in serum [29]. In most studies, rectal cultures were obtained before administrating antibiotic prophylaxis [7, 9, 12, 13, 15–17, 20, 22–25]. In some studies however, rectal cultures were taken after antibiotic (AB) prophylaxis was administered [8, 10, 11, 14, 15, 17–19, 21]. In this last group, antibiotics might already be present in the rectal mucosa when rectal cultures were taken which could influence prevalence rates by selecting FQ-R bacteria. We therefore advise taking rectal cultures before administrating prophylactic antibiotics in order to adequately determine prevalence rates.

When determining the population that benefits most from targeted therapy, it is important to identify risk factors. Ideally, rectal culture processing and the application of targeted therapy should be reserved for patients with a high risk of having FQ-R bacteria. Several studies that were incorporated in our review have examined one or more risk factors. Previous antibiotic exposure was clearly the most important. An explanation for the higher prevalence of FQ-R bacteria after prior FQ use was proposed by Horcajada et al. who stated that a previous negative rectal culture for FQ-R* E. coli* might become positive by selecting low numbers of FQ-R bacteria in a heterogenic bacterial population or following a shift of intermediate sensitive strains to fully resistant strains by antibiotic pressure [30]. However, how long the use of FQ antibiotics persists as a risk factor for the carriage of FQ-R bacteria in the rectum remains unclear. The definition of previous antibiotic use differs greatly between the 5 studies that defined this parameter as a risk factor (3 months [11, 14], 6 months [10], and up to 5 years [9, 20]). We advise the documentation of a 6-month period based on evidence from Yacgi et al. in 2009 who reported that FQ use in the last 6 months was an important risk factor for FQ-R carriage [31].

A second important risk factor for having FQ-R rectal bacteria is a history of previous prostate biopsies. This risk factor was responsible for a not significant difference in two studies [8, 9] and a significant difference in one study [23]. A possible explanation could be that these patients also received prophylactic FQ antibiotics during biopsy. Obviously, more research needs to be conducted to identify the patient groups who are at high risk of FQ-resistance and may benefit from patients targeted therapy.

This review showed an infectious complication rate of 7.9% in patients receiving prophylactic FQ and harbouring FQ-R bacteria compared to 1.6% in patients not harbouring FQ-R bacteria. This means a fivefold higher risk when no adapted prophylaxis is given. It remains unsure/doubtful if this also represents a statistically significant difference, due to large demographical and geographical differences between the studies. In the targeted therapy group, there was only a small difference in the rate of infectious complications between the targeted unmodified and targeted modified groups (0.5% and 0.8%). This means that, despite an accurate antibiotic policy, infectious complications can still develop. There are two possible explanations for this finding. A first possibility is that antibiotic susceptibility testing was not completely accurate or that prophylactic antibiotics were not correctly applied (due to medical error or noncompliance of the patient). A second and more plausible possibility is that host and procedural factors that can significantly influence the risk of infectious complications possibly exist and remain to be elucidated.

The ultimate question regarding targeted prophylaxis based on rectal cultures is whether this approach reduces the risk of infectious complications. When studies with a targeted approach were prospectively compared to results from studies with an empirical approach (without rectal cultures) only minor differences were seen (0.6% complications in the targeted group versus 0.8% complications in the empirical group) [13, 22, 24, 25]. This minor difference might be explained by 2 major limitations of the current studies (Table 2).

We believe underreporting of infectious complications is a major issue which makes it difficult to prove that targeted therapy has better results than empirical therapy. Underreporting can occur because of several circumstances. Firstly some of these studies only assess severe infectious complications such as sepsis. Minor infections complications such as prostatitis, epididymitis, pyelonephritis, and uncomplicated urinary tract infections (UTIs) are much more frequent than a urinary sepsis or septic shock [2]. In a study performed by Suwantarat et al., rectal cultures were taken in a targeted and in an empirical group, thereby ignoring the results of the rectal cultures in this last group. This study reported not only sepsis but also minor infectious complications such as UTIs and reported 9% complications in the empirical therapy group compared to 0.5% complications in the targeted therapy group [17]. Secondly a post-TRPB observation period that is too short could also cause underreporting of infectious complications. Most studies use a 30-day follow-up period [7, 13, 15, 17, 21–25]. However, some studies limited this follow-up period to 7 days or 14 days [8, 9, 11, 12, 14, 18, 20]. Although most complications appeared in the first days following the biopsy, UTIs were sometimes seen after 14 days. Therefore, we recommend a follow-up period of at least 30 days.

Thirdly an inadequate registration method could underreport infectious complications. We advise the use of direct patient contact through a telephone call or a follow-up consultation. This is a time-consuming method but is probably more accurate than the use of electronic medical records (EMR) or the retrievement of surveillance urinary samples in the identification of infectious complications.

The absence of randomisation is the second major limitation of studies comparing targeted versus empirical therapy. Often the decision to take rectal cultures was made at the discretion of the practitioner. This implies that patient selection may be not unequivocal: patients with multimorbidity and a higher risk of developing infectious complications might more often receive targeted therapy because the practitioner does not want to induce infections in these frail patients.

One study by Taylor et al. performed a cost analysis of the targeted therapy approach. They compared the costs per person of a group that received empirical antibiotic prophylaxis to a group that received targeted prophylaxis as part of a study protocol. It was calculated that targeted therapy was cost-efficient in their study population since the group with targeted prophylaxis did not have any infectious complication (0/112) and the group with empirical prophylaxis had infectious complications (9/345). The number needed to treat to prevent one infectious complication was 38 and a total of $4499 was gained per averted infectious complication [13]. However, these results cannot be extrapolated to other studies. The local prevalence of FQ-R bacteria will certainly play a pivotal role in the calculation whether targeted therapy is cost-efficient in a certain region. Data from Hanna et al. in Norwich, UK, showed very low prevalence rates of FQ-R bacteria (4%) meaning that targeted prophylaxis will probably not be useful and certainly not cost-efficient in this population [21]. Moreover the cost of medical care is variable and should be calculated individually.

## 5. Conclusion

The global prevalence of rectal FQ-R bacteria prior to TRPB is high and FQ-resistance is still increasing due to overuse of this group of antibiotics. Current studies on the use of rectal cultures in the prebiopsy setting have too many limitations and confounding variables to definitely accept this approach in clinical practice. Whether this methodology is useful in a certain region will greatly depend on local FQ-R prevalence rates. Also, well-constructed randomised and blinded studies are warranted in order to obtain sufficient evidence to document a reduction of infection rates. Risk factors need to be better defined to narrow the group that benefits from this approach in order to achieve a high cost-effectiveness ratio.

## Figures and Tables

**Table 1:** Comparing infectious complications after prostate biopsy in patients with fluoroquinolone-resistant and fluoroquinolone-sensitive rectal cultures. (Empirical therapy.)

Infectious complications after prostate biopsy in patients receiving				
empirical FQ prophylaxis with FQ-resistant and FQ-sensitive rectal cultures				
Author	FQ-R on RC	ICs in FQ-R	FQ-S on RC	ICs in FQ-S
Batura et al., 2010 [7]	47	7	398	1
Liss et al., 2011 [8]	30	1	106	4
Minamida et al., 2011 [9]	13	4	87	0
Steensels et al., 2012 [10]	58	7	178	0
Siriboon et al., 2012 [11]	133	2	11	0
Taylor et al., 2013 [14]	161	15	696	16
Liss et al., 2013 [15]	18	0	82	0
Suwantarat et al., 2013 [17]	21	9	111	3
Lee et al., 2014 [19]	25	3	100	1
Tsu et al., 2015 [20]	150	5	221	4
Hanna et al., 2015 [21]	10	0	257	7
		8% ICs if FQ-R		1.6% ICs if FQ-S
		bacteria in RC		bacteria in RC

**Table 2:** Limitations of studies examining targeted therapy for prostate biopsies.

Study bias	Risk	Advice
Not reporting minor infectious complications	Underreporting of infectious complications	Reporting of prostatitis, epididymitis, pyelonephritis, uncomplicated urinary tract infections, and sepsis and septic shock
Insufficient length of follow-up		Follow-up period of at least 30 days
Inadequate registration method		Direct patient contact through a telephone call or follow-up consultation

Practitioner decides in which patient rectal cultures are taken	Biased study population: risk of excluding frail patients	Prospective randomisation after obtaining informed consent
